# Attentional Prioritization of Infant Faces Is Limited to Own-Race Infants

**DOI:** 10.1371/journal.pone.0012509

**Published:** 2010-09-01

**Authors:** John Hodsoll, Kimberly A. Quinn, Sara Hodsoll

**Affiliations:** 1 School of Biological and Chemical Sciences, Queen Mary University of London, London, United Kingdom; 2 School of Psychology, University of Birmingham, Birmingham, United Kingdom; 3 Department of Psychology, University College London, London, United Kingdom; Kyushu University, Japan

## Abstract

**Background:**

Recent evidence indicates that infant faces capture attention automatically, presumably to elicit caregiving behavior from adults and leading to greater probability of progeny survival. Elsewhere, evidence demonstrates that people show deficiencies in the processing of other-race relative to own-race faces. We ask whether this other-race effect impacts on attentional attraction to infant faces. Using a dot-probe task to reveal the spatial allocation of attention, we investigate whether other-race infants capture attention.

**Principal Findings:**

South Asian and White participants (young adults aged 18–23 years) responded to a probe shape appearing in a location previously occupied by either an infant face or an adult face; across trials, the race (South Asian/White) of the faces was manipulated. Results indicated that participants were faster to respond to probes that appeared in the same location as infant faces than adult faces, but only on own-race trials.

**Conclusions/Significance:**

Own-race infant faces attract attention, but other-race infant faces do not. Sensitivity to face-specific care-seeking cues in other-race kindenschema may be constrained by interracial contact and experience.

## Introduction

Human (and non-human) infants are wholly dependent for their survival on a parent or caregiver. Given this dependence, a mechanism by which infants can automatically attract caregiving behavior would promote their survival [1]. Lorenz coined the term *kindenschema* (“baby schema”) to describe one possible mechanism of this sort: a set of key features found in infant faces of virtually all species, including big eyes, a large and high forehead, rounded cheeks, and a small nose and mouth [2]. Indeed, stimuli that conform to this baby schema are likely to elicit a positive behavioral response [3], [4], and the perception of infant features activates brain structures associated with the reward system [5], [6]. Baby schemas appear to draw out the kinds of responses that would motivate caregiving behavior.

A consequence of a caregiving instinct is that baby schema should not only elicit positive responses, but may also receive attentional priority over the processing of other stimuli. Attentional prioritization of infants would enhance infant-caregiver interactions by facilitating caregivers' ability to detect and respond to signs of emotional distress in the infant [7]. Evidence for just these effects on attention has been shown in two studies by Brosch and colleagues [8], [9]. Attentional capture by infant faces was shown in a spatial probe detection task; probe detection at locations primed by infant faces was facilitated relative to locations primed by adult faces.

In the current experiment, we investigated whether the preferential allocation of attention to infant faces would be influenced by the race of the faces and of the perceivers viewing them. A large body of research has shown that people show deficiencies in the processing of other-race relative to own-race faces. Specifically, people are better at discriminating, recognizing, and detecting changes in own-race faces than other-race faces [**10**]–[**12**]. This “other-race effect” (ORE) appears to have an expertise component, such that people may be less sensitive to the facial cues that individuate members of other racial groups [13], [14], and/or less likely or able to use the holistic processing that supports own-race face individuation and recognition in the processing of other-race faces [15], [16]. Indeed, people with greater exposure to other-race individuals manifest weaker OREs, if they manifest them at all [17]. In addition, however, the ORE also appears to have a motivational component, such that processing of other-race but not own-race faces is truncated at the point where a race-specifying cue (e.g., skin tone) is detected in a face [18]. Irrespective of which (if either) mechanism is more responsible for the emergence of OREs, both accounts suggest that OREs result from the failure to differentiate among individual members of other-race categories.

The implications of this tendency to see other-race faces as interchangeable with each other are unclear in the context of attentional capture by kindenschema. On the one hand, the biological significance of kindenschema should guarantee that infant faces of any race capture attention; in this case, both own- and other-race infant faces would be expected to capture attention. On the other hand, the robustness of OREs raises the intriguing possibility that other-race infant faces, like other-race adults faces, may not be individuated effectively; in this case, only own-race infant faces would be expected to capture attention.

**Figure 1:**
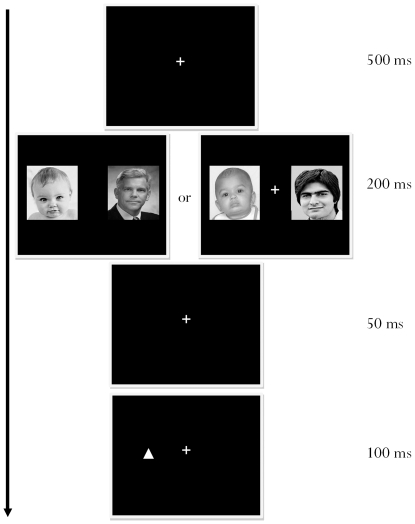
Trial sequence for probe detection task (White infant–adult face pairing and South Asian infant–adult pairing).

To examine this issue, we used a version of a standard probe-detection task to measure the allocation of spatial attention [8], [19]. Two stimuli—here, faces of different age categories (infant and adult)—were flashed simultaneously on the computer screen, each flanking a centrally placed fixation cross (see Figure 1); importantly, half of the face pairs were South Asian and half were White. South Asian and White participants viewed the face pairs and, for each pair, reported the orientation of a probe shape that appeared at either the location previously occupied by the infant face or the location previously occupied by the adult face.

## Results

A 2 (face age at probe location: infant, adult) ×2 (face race at probe location: South Asian, White) ×2 (participant race: South Asian, White) mixed-model analysis of variance (ANOVA) indicated that probe Reaction Times (RTs) were faster when probes appeared in the same location as infant faces (*M* = 513 ms) than adult faces (*M* = 524 ms), *F*_1,38_ = 13.31, *P* = 0.001. Importantly, however, the infant-face advantage depended on distractor race and participant race, *F*_1,38_ = 4.54, *P* = 0.04.

**Figure 2:**
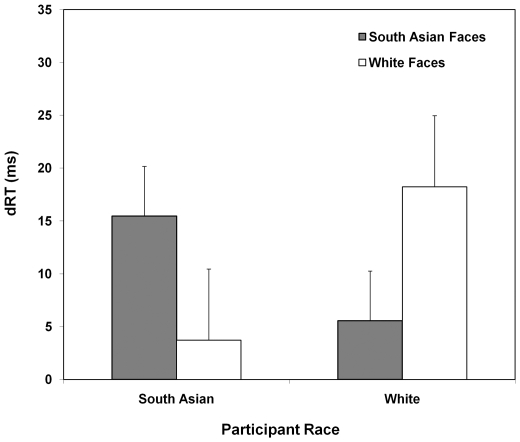
Performance on probe detection task. Difference RTs for probes appearing behind adult faces minus probes appearing behind infant faces, as a function of stimuli and participant race. *Note.* Higher numbers indicate greater attentional allocation to infant over adult faces. Error bars represent ± 1S.E.

Figure 2 depicts this Probe Location Age x Probe Location Race x Participant Race interaction in terms of difference RTs (RTs to probes at adult-face locations minus RTs to probes at infant-face locations; note that absolute RTs are shown in Table 1). Paired *t*-tests confirmed that infant faces elicited faster probe RTs than adult faces only if they matched the race of the participant: South Asian participants responded to probes more quickly when they appeared in the same location as infant face than adult face distractors for South Asian infant–adult face pairs, *t*_19_ = 2.63, *P* = 0.016, but not for White infant–adult face pairs, *t*_19_ = 0.46, *P*>0.05. White participants showed the opposite pattern, responding to probes more quickly when they appeared in the same location as infant face than adult face distractors for White infant–adult face pairs, *t*_19_ = 3.54, *P* = 0.002, but not for South Asian infant–adult face pairs, *t*_19_ = 1.67, *P*>0.05. (A similar analysis on error rates showed no significant effects, all *P*s>0.5, confirming that the RT pattern cannot be explained in terms of a speed–accuracy trade-off; see Table 2.)

**Table 1:** Mean probe-detection reaction times (±1 standard error) in milliseconds as a function of distractor age, distractor race, and participant race; experimental study (64 trials per condition).

	South Asian Participants		White Participants	
	South Asian Distractors	White Distractors	South Asian Distractors	White Distractors
Infant distractors	516	528	512	495
	(20.1)	(21.6)	(21.0)	(21.6)
Adult distractors	532	532	518	514
	(21.9)	(20.7)	(21.9)	(20.7)

**Table 2:** Mean probe-detection error rates (±1 standard error) as a function of distractor age, distractor race, and participant race; experimental study (64 trials per condition).

	South Asian Participants		White Participants	
	South Asian Distractors	White Distractors	South Asian Distractors	White Distractors
Infant distractor	10.0	10.1	9.1	9.3
	(1.2)	(1.3)	(1.2)	(1.3)
Adult distractors	10.0	10.3	9.5	9.1
	(1.2)	(1.3)	(1.2)	(1.3)

## Discussion

These results suggest that the attentional prioritization of kindenschema is modulated by the race of the infant and of the perceiver. In the current experiment, infant faces captured attention only when they matched the race of the participant. This was true for both South Asian and White participants, showing that this pattern of data was not due to perceptual artifacts in the stimuli. To what, then, can we attribute the effects?

One possibility is simply that other-race infant faces, like other-race adults faces, tend not to be processed holistically, and thus that they were not individuated from adult faces in the current study. This lack of individuation might have resulted because the detection of a racial cue truncates processing [13], [18]. Simple cues such as skin tone are sufficient for racial category to be recognized [**20**]–[**22**], but perceiving kindenschema (e.g., big eyes, small nose, large forehead) requires more configural processing; that is, features can only be recognized as large or small in the context of the entire face/head. Assuming the scale of face perception proceeds across time from the extraction of simpler features (e.g., skin tone to determine race) to more complex configurations (e.g., size of eyes within the face) [**23**]–[**26**], then early social categorization effects might moderate the influence of kindenschema.

Another possibility is that participants in the current study lacked the expertise to individuate other-race faces as efficiently as own-race faces. It is also possible that this relative inability to individuate other-race faces was confined primarily to other-race *infant* faces. The study was conducted in an ethnically diverse city (with almost 20% of the population self-identifying as South Asian) and all of our participants were UK-born and thus were likely to have had a reasonable amount of interracial contact, particularly our minority South Asian participants. Given our participants' age (19–20 years) and status (undergraduate university students), however, it is conceivable that their prior interracial experiences were with other-race adults more than with other-race infants.

This is not to say our participants failed to recognize infant faces as such; it is merely to suggest that factors that undermine individuation (i.e., social categorization, lack of experience) might also offset the effects of kindenschema. Another factor, however, might be the relative differences in arousal engendered by own-race versus other-race adult faces. Brosch and colleagues [8], [9] suggested that infant faces capture attention because their biological significance makes them more arousing—and thus more salient—than other categories of stimuli. Differential positivity or arousal associated with infant versus adult faces alone cannot account for the attentional prioritization of infant faces in the current data, however: Within-race comparisons indicated that both own- and other-race infant faces were rated as more pleasant and more arousing than their adult counterparts in pilot testing (see Table 3), and yet attentional capture by infant faces only occurred for own-race faces. Interestingly, however, between-race comparisons indicated that other-race adult faces were rated as more arousing than own-race adult faces by both South Asian and White participants (*t*_7_ = 4.85 *P*<0.001 and *t*_7_ = 3.29 *P* = 0.013, respectively). It may be that within the context of viewing both own- and other-race faces, the greater arousal level associated with other-race adult faces was sufficient to interfere with the attentional prioritization of other-race infant faces, despite other-race infant faces being rated as more arousing than their adult counterparts. This possibility is the subject of ongoing investigation.

**Table 3:** Mean pleasantness and arousal ratings (±1 standard error) as a function of target age, target race, and participant race in the pilot study; possible range  = 0 to 100.

		South Asian Participants		White Participants	
		South Asian Distractors	White Distractors	South Asian Distractors	White Distractors
Pleasantness					
	Infant faces	81.2	77.1	67.4	83.5
		(0.7)	(0.9)	(0.7)	(0.9)
	Adult faces	35.3	28.3	35.4	42.0
		(3.3)	(2.4)	(3.3)	(2.4)
	*P*-value (infant versus adult)	0.001	0.001	0.001	0.001
Arousal					
	Infant faces	79.3	84.2	69.6	78.2
		(1.1)	(1.5)	(1.1)	(1.5)
	Adult faces	18.3	28.0	42.8	24.6
		(2.8)	(2.1)	(2.8)	(2.1)
	*P*-value (infant versus adult)	0.001	0.001	0.001	0.001

*Note.* The *P* value refers to a *t*-test comparing the infant and adult faces per column condition (e.g., comparing ratings of South Asian adult and infant faces by South Asian participants).

In providing evidence that attentional capture by infant faces is modulated by the race of the faces and of the perceivers viewing them, these results also suggest a qualification of the cooperative breeding hypothesis [27]. According to this hypothesis, infant caregiving is distributed across members of the species (kin and non-kin) rather than the responsibility of the parents alone—a form of caregiving called allo-parenting. This notion is compatible with Brosch and colleagues' (8) evidence for attentional prioritization of human but not non-human infant faces. Our results, however, suggests that the attentional filter imposed by this presumably evolutionary drive toward allo-parenting is tuned to the individual's environment and experience. For those without frequent interracial contact, particularly with other-race infants, the attentional filter may operate more efficiently at the level of social category rather than species: In the absence of sufficient motivation [28], [29], expertise [30], [31], or training [32], [33] to individuate other-race infant faces, these individuals may be less sensitive to face-specific care-seeking cues such as kindenschema when those cues are emitted by other-race versus own-race infants.

## Materials and Methods

### Participants and Design

Participants were 20 South Asian and 20 White female UK-born undergraduates at the University of Birmingham (mean ages 20.25 years and 19.63 years, respectively). All participants were right-handed, had normal or corrected-to-normal vision, and received course credits for their participation. The experiment was based on a 2 (probe location age: infant, adult) ×2 (probe location race: South Asian, White) ×2 (participant race: South Asian, White) mixed design, with probe location age and race as within-participants factors.

### Apparatus and Stimuli

The task was performed on an IBM-compatible PC using a Philips 19” CRT monitor. Stimulus presentation and response collection were managed with E-Prime v1.20 (Psychology Software Tools, Pittsburgh, USA; www.pstnet.com/eprime).The stimuli comprised a probe shape and face images. The probe shape was a small equilateral triangle of RGB value (160, 160, 160) subtending 0.8 cm. Face images, downloaded from the internet, were grayscale portrait images of South Asian and White infants and middle-aged adults (eight in each Age x Race category), all showing neutral to slightly positive facial expressions. The adult stimulus category comprised four female and four male faces. The images were formatted to be similar in terms of contrast, sharpness, brightness, and any salient features. The images were 6 cm ×6 cm in size and were presented at 12 cm to the left or right of the fixation cross, giving a visual angle of at 70 cm distance from the screen.

For the experimental task, the faces images were presented in pairs. Each pair comprised an infant face and an adult face of the same race; that is, South Asian infant faces were paired with South Asian adult faces and White infant faces were paired with White adult faces. Assignment of infant and adult faces to the left and right sides of the display was counterbalanced across trials. Note that there were differences between the infant and adult face stimuli other than the presence/absence of kindenschema. In particular, there were clear clothing and hairline cues in the adult faces that were not present in the infant faces. Nonetheless, these differences cannot account for the pattern of findings whereby attentional prioritization of infant over adult faces occurred for own- but not other-race faces, as the same age confound was present in both South Asian and White face stimuli.

### Procedure

Prior to participation, participants completed consent forms and a personal information sheet on which they indicated their ethnic origin. They then completed a probe-discrimination task. At the start of each trial, a fixation cross appeared in the centre of the screen for 500 ms. Next, two faces images appeared for 200 ms, one on each side of the fixation cross. After a 50-ms interval, a small triangle appeared for 100 ms at the centre of one of the previous image locations, followed by a blank screen until the participant's response. Participants indicated whether the probe triangle pointed upwards or downwards by pressing the respective “J” and “N” keys on the computer keyboard. Once a response was recorded, the next trial began. Following a set of 32 practice trials designed to familiarize participants with the task, participants completed 2 blocks of 128 fully randomized trials, giving 64 trials per experimental condition. Upon completion of the task, participants were thanked and informed about the purpose of the experiment.

### Statistical Analysis

Mean response times (RTs) to probes served as the dependent measure of interest. RT outliers were removed using a recursive procedure with a moving criterion [34]. The remaining mean correct probe RTs were entered into a 2 (probe location age: infant, adult) ×2 (probe location race: South Asian, White) ×2 (participant race: South Asian, White) analysis of variance (ANOVA).

### Pilot Study

A preliminary study was conducted where eight South Asian participants and eight White participants (all female undergraduate students) rated the 32 selected stimuli (see *Apparatus and Stimuli*, below) in terms of pleasantness and arousal. The stimuli were presented amongst 16 additional grayscale images, eight of which were flowers and eight of which were world leaders and politicians (of different races). The different categories of images were scrambled and presented separately on a computer screen using Microsoft PowerPoint. For pleasantness ratings, participants rated how warm they felt towards each image by indicating a particular temperature on a thermometer ranging from 0° (*no warm feelings*) to 100° (e*xtremely warm feelings*). For arousal ratings, participants rated the same images according to how each image made them feel, along a similar scale ranging from 0 (*not alert at all*) to and 100 (*very alert*).

We entered the pleasantness rating scores into a mixed-model ANOVA with target age and target race as within-participants factors and participant race as a between-participants factor (see Table 3). Overall, infant faces were rated as more pleasant than adult faces, *F*_1,14_ = 535.48, *P* 0.001, South Asian faces were rated as more pleasant than White faces, *F*_1,14_ = 9.81, *P*<0.007, and own-race faces were rated as more pleasant than other-race faces, *F*_1,14_ = 81.25, *P*<0.001. In addition, the preference for infant over and adult faces was greater for own-race than cross-race faces for White participants but not South Asian participants, *F*_1,14_ = 8.453, *P* = 0.011. Of most import for this study, infant faces were rated as significantly more pleasant than adult faces regardless of target race or participant race, all *P*<0.001.

Arousal ratings were subjected to the same analysis (Table 3). Overall, infant faces were rated as more arousing than adult faces, *F*_1,14_ = 985.27, *P*<0.001. All interactions were significant: Target Race x Participant Race, *F*_1,14_ = 24.62 *P*<0.001; Target Age x Participant Race, *F*_1,14_ = 33.95, *P*<0.001; Target Age x Target Race, *F*_1,14_ = 41.01, *P*<0.001; and Target Age x Target Race x Participant Race, *F*_1,14_ = 50.59, *P*<0.001. Although both South Asian and White participants found White infant faces more arousing than South Asian infant faces, the pattern of data differed for adult faces: Both South Asian and White participants found other-race adult faces more arousing than own-race adult faces. Critical to the current experiment, infant faces were rated as significantly more arousing than adult faces, regardless of target race or participant race, all *P*<0.005.
